# Yellow fever impact on brown howler monkeys (*Alouatta guariba clamitans*) in Argentina: a metamodelling approach based on population viability analysis and epidemiological dynamics

**DOI:** 10.1590/0074-02760150075

**Published:** 2015-11

**Authors:** Eduardo S Moreno, Ilaria Agostini, Ingrid Holzmann, Mario S Di Bitetti, Luciana I Oklander, Martín M Kowalewski, Pablo M Beldomenico, Silvina Goenaga, Mariela Martínez, Eduardo Lestani, Arnaud LJ Desbiez, Philip Miller

**Affiliations:** 1 Universidade Federal do Oeste do Pará, Programa de Pós-Graduação Natureza, Sociedade e Desenvolvimento, Santarém, PA, Brasil; 2 Universidad Nacional de Misiones, Consejo Nacional de Investigaciones Científicas y Técnicas, Instituto de Biología Subtropical,Puerto Iguazú, Misiones, Argentina; 3 Centro de Investigaciones del Bosque Atlántico, Puerto Iguazú, Misiones, Argentina; 4 Museo Argentino de Ciencias Naturales Bernardino Rivadavia, Consejo Nacional de Investigaciones Científicas y Técnicas, Estación Biológica de Corrientes, San Cayetano, Corrientes, Argentina; 5 Universidad Nacional del Litoral, Consejo Nacional de Investigaciones Científicas y Técnicas, Instituto de Ciencias Veterinarias del Litoral, Laboratorio de Ecología de Enfermedades, Esperanza, Santa Fe, Argentina; 6 Instituto Nacional de Enfermedades Virales Humanas Dr Julio I Maiztegui, Pergamino, Buenos Aires, Argentina; 7 Instituto Nacional de Medicina Tropical, Puerto Iguazú, Misiones, Argentina; 8 Royal Zoological Society of Scotland, Edinburgh, Scotland, UK; 9 International Union for Conservation of Nature, Species Survival Commission, Conservation Breeding Specialist Group, Apple Valley, MN, USA

**Keywords:** conservation medicine, wildlife disease, disease impact, sensitivity analysis

## Abstract

In South America, yellow fever (YF) is an established infectious disease that has been identified outside of its traditional endemic areas, affecting human and nonhuman primate (NHP) populations. In the epidemics that occurred in Argentina between 2007-2009, several outbreaks affecting humans and howler monkeys (*Alouatta* spp) were reported, highlighting the importance of this disease in the context of conservation medicine and public health policies. Considering the lack of information about YF dynamics in New World NHP, our main goal was to apply modelling tools to better understand YF transmission dynamics among endangered brown howler monkey (*Alouatta guariba clamitans*) populations in northeastern Argentina. Two complementary modelling tools were used to evaluate brown howler population dynamics in the presence of the disease: Vortex, a stochastic demographic simulation model, and Outbreak, a stochastic disease epidemiology simulation. The baseline model of YF disease epidemiology predicted a very high probability of population decline over the next 100 years. We believe the modelling approach discussed here is a reasonable description of the disease and its effects on the howler monkey population and can be useful to support evidence-based decision-making to guide actions at a regional level.

Yellow fever (YF) is an infectious disease caused by YF virus (YFV), a member of the family Flaviviridae. In South America, the YFV is endemic to rainforests where it persists, infecting canopy-dwelling mosquitoes, such as *Haemagogus* and*Sabethes* (Diptera: Culicidae) and nonhuman primates (NHP), in what has been termed "sylvatic cycle" (Vasconcelos et al. 2004). A few studies demonstrated that YF is endemic to some regions of South America (Vasconcelos 2010), in the Orinoco and Araguaia regions (Barrett & Higgs 2007) and, especially, the Amazon Basin (Souza et al. 2010). From those foci, epidemic waves of viral dissemination tend to occur in cycles of between seven-14 years (Vasconcelos et al. 2004,Vasconcelos 2010, Camara et al. 2011). The periodicity of viral dissemination has been suggested to be linked with NHP population dynamics. In particular, with the availability of new susceptible individuals in the populations, which are essential for viral amplification (Hervé & Travassos da Rosa 1983, Monath 1989).

NHP species show different levels of susceptibility to the disease (Kumm & Laemmert 1950, Hervé & Travassos da Rosa 1983, Thoisy et al. 2004). In particular, the genus *Alouatta* (howler monkeys) seems to be the most susceptible of all NHP (Araújo et al. 2011). These animals show acute forms of the disease, with severe clinical evolution and high mortality (Crockett 1998, Sallis et al. 2003, Holzmann et al. 2010, Moreno et al. 2013). For this reason, howler monkeys are considered excellent sentinel species for the early detection of YF epidemics (Araújo et al. 2011). During the periods 2000-2003 and 2007-2010, the circulation of YFV in South America was identified in areas considered for decades to be free of the disease, being detected in southeastern and southern Brazil in 2000 (Vasconcelos et al. 2001)and again in 2007 (Araújo et al. 2011, Moreno & Barata 2011) and hitting northeastern Argentina in November 2007 (Holzmann et al. 2010,Goenaga et al. 2012). In Misiones province in northeastern Argentina, YF was diagnosed in howler monkeys and humans (Goenaga et al. 2012). In Argentina during 2007 and 2008, YF outbreaks killed at least 59 howler monkeys, decimating these primates throughout their southern distribution (Bicca-Marques & Freitas 2010, Cardoso et al. 2010, Holzmann et al. 2010, Goenaga et al. 2012).

Two howler monkey species occur in Argentina: the black-and-gold howler monkey (*Alouatta caraya*) and the southern brown howler monkey (*Alouatta guariba clamitans*). The brown howler is the rarest primate species in Argentina, restricted to east-central Misiones province (Holzmann et al. 2010). Due to the likely significant impact of these epidemics, there is special concern about the current status of this specie. In Argentina, the brown howler has been re-classified from "endangered" to "critically endangered" (Agostini et al. 2012). In order to establish conservation priorities for this species and its habitat in Argentina, an assessment of the current brown howler population status and the main threats to the specie persistence was carried out in the Karadya Bio-Reserve near Comandante Andresito and Puerto Iguazú (Misiones). A group of nine experts in different fields (primate ecology, eco-epidemiology, mosquito ecology and virology) dedicated themselves to gathering, systematising and discussing all available data and information on brown howlers and YF in the Atlantic Forest. Agostini et al. (2014) showed all the steps to prioritise objectives and actions for the development of a Species Conservation Strategy in Argentina. Our goal in this paper is to present, in a more detailed fashion, the combined use of different modelling tools to simulate YFV transmission dynamics among brown howler monkey populations in northeastern Argentina in order to gain a better understanding of YF dynamics in New World NHP. We also describe the process of definition of the input parameters, highlighting the contribution of each one for the whole system.

## MATERIALS AND METHODS

We estimated input parameter values and constructed simulation scenarios for two modelling tools commonly used in International Union for Conservation of Nature/ Species Survival Commission - Conservation Breeding Specialist Group workshop processes: Vortex (Lacy & Pollak 2013) and Outbreak (Lacy et al. 2012). Vortex is a Monte Carlo simulation for population viability analysis that examines the effects of deterministic forces as well as demographic, environmental and genetic stochastic events on wild population abundance and growth dynamics. Demographic information such as breeding and mortality rates for general sex-specific stages (juveniles, sub-adults and adults) is user-specified and used to project total population size. Outbreak is a software package that simulates susceptible, exposed, infectious or recovered/resistant (SEIR)-style disease dynamics using the basic conceptual algorithms of Anderson (1982) and May (1986) as a foundation. Using this approach, individuals are classified as SEIR.

To model infectious processes, the state of each individual in the population is tracked and the probabilities of transition among states are specified as functions of the number of individuals in each state and of other relevant parameters, such as the contact rate and the latent period of infection. To make the software able to model the transmission of a vector-borne disease, we conducted basic adaptations in some specific components (see *Input parameters for stochastic disease epidemiology models*).

*General approach for model construction* - We decided to use the advantages of both Vortex and Outbreak to create a more realistic model representing the dynamics of YF in brown howler populations in Argentina. To do this, we used a new technology called "metamodelling" (Miller & Lacy 2003, Bradshaw et al. 2012,Lacy et al. 2013). A metamodel is composed of two or more independent, discipline-specific models that exchange data in order to reveal emergent dynamic properties of a complex system. In this approach, the output of one model can modify inputs to another model (e.g., Lacy et al. 2013, Medina-Miranda et al. 2013). This metamodel approach, utilising the complementary strengths of each modelling tool, allows us to analyse the population-level impacts of simulated YF outbreaks in a more detailed and realistic fashion compared to methods using each software alone.

To implement metamodelling we followed Lacy et al. (2013). We built a generic platform, MetaModel Manager (Lacy & Pollak 2013), a software that links simulations of one or more populations with any number of additional "modifier" models that create, use and modify characteristics of individuals, populations or environments (Lacy et al. 2013). In our metamodel, population viability was predicted using the Vortex, while the disease epidemiological dynamics were simulated using Outbreak.

In order to evaluate the robustness of the model to parametric uncertainty, a sensitivity analysis was conducted. In general, the sensitivity of a given model input parameter measures the proportional change in a given output parameter (e.g., stochastic population growth rate) that results from a given proportional change in the input parameter. In the current context, this analysis was used to uncover particularly sensitive parameters that could significantly alter the results and conclusions derived from the model. In all models the sensitivity analysis was conducted using alternative values for variables related to demographic parameters of the howler monkeys (e.g., reproductive age, natural mortality rates), as well epidemiological parameters (e.g., viral incubation periods, contact rates).

*Input parameters for Vortex demographic model* - A general baseline population model for brown howler monkeys was built and it was later tailored to represent the populations of Misiones. The baseline population model was designed to investigate the viability of a nonexistent but biologically accurate howler population without any anthropogenic threats. The baseline model reflects the biological potential of brown howlers. Alternative values for demographic parameters were then explored through sensitivity testing.

*Scenario settings* - *Duration of simulation* - Life expectancy of howlers is approximately 10-18 years in the wild. The population was modelled for 100 years (approximately 15 generations) so that long-term population trends could be observed. One hundred years is far enough into the future so as to decrease the chances of omitting a yet unknown event, but also not too short to fail to observe a slowly developing event.

*Number of iterations* - One thousand independent iterations were run for each scenario.

*Species description* - *Definition of extinction* - Extinction was defined in the model as no animals of one or both sexes remain.

*Concordance of environmental variation (EV) between reproductive rates and survival rates* - EV is the annual variation in reproduction and survival due to variation in environmental conditions. Making EV concordant between reproduction and survival means that good years for reproduction are also good years for survival and *vice versa*.

In Vortex the EV is modelled by the user which specifies a mean and a standard deviation (SD) values for each rate. The EV is determined by solving the equation for the binomial variance,* V = p(1-p)/N*, for the parameter*N* when given the mean, *p* and variance,*V = SD^2^*(Lacy et al. 2013). In our model, we estimated the SD of baseline values for some parameters like "annual % adult females reproducing" and "% mortality from different ages" (Table I).

**TABLE I:** Summary of parameter input values used in the Vortex baseline model

Parameter	Baseline value	Alternate values
Populations (n)	1	-
Initial population size	200	-
Carrying capacity	420	-
Inbreeding depression	6 LE	-
Effect of inbreeding due to recessive lethal alleles (%)	50	-
Breeding system	Long-term polygyny	-
Age of first reproduction (♀/♂)	5/6	4/5
Maximum age of reproduction	16	13/20
Annual adult females reproducing [% (SD)]	50 (22)	-
Average litter size	1	-
Density dependent reproduction?	No	-
Maximum litter size	1	-
Overall offspring sex ratio	50:50	-
Adult males in breeding pool	90	-
Mortality {age (♀/♂) [SD (%)]}		
0-1	25 (5)/25 (5)	-
1-2	15 (5)/15 (5)	-
2-3	10 (2.5)/10 (2.5)	-
3-4	5 (2)/5 (2)	-
4-5	2 (1)/10 (2.5)	-
5-6	1 (0.5)/10 (1)	-
6-10	1 (0.5)/1 (0.5)	-
11-16	+5% each year/+20% each year	-

LE: lethal equivalent; SD: standard deviation.

*Inbreeding depression* - Wild populations that live in potentially more challenging environments are more vulnerable to inbreeding (Crnokrak & Roff 1999, O'Grady et al. 2006). In this case vulnerability is related to individuals with relatively low allozyme heterozygosity and/or with a high number of lethal equivalent (LE) alleles and so, are much more susceptible to factors that may not affect "normal" individuals (Crnokrak & Roff 1999). Environmental factors such as unpredictable rainfall, fluctuating temperatures and limiting resources to feed young are all likely to have a significant effect on juvenile mortality in general (Ralls et al. 1988).

Crnokrak and Roff (1999) examined 157 datasets for wild populations of 34 taxa and found that 90% showed evidence of inbreeding depression. The median value estimated from analysis of studbook data for 40 captive mammal populations was 3.14 LE (Ralls et al. 1988). O'Grady et al. (2006) found an average overall effect of 12.3 LE over the life history of wild mammal and bird populations, with 6.3 LE impacting the production and survival of offspring to age one year. Based on these studies, the impact of inbreeding was modelled as 6 LE on juvenile mortality, with 50% of the effect of inbreeding due to recessive lethal alleles.

*Reproductive system and rates - Breeding system [long term polygyny (Mitani et al. 1996)] - Age of first reproduction* - Vortex defines reproduction as the time at which offspring are born, not simply the age of sexual maturity. The program uses the mean age of first reproduction rather than the earliest recorded age of reproduction. In*A. caraya*, females reach sexual maturity around three-four years (Raguet-Schofield & Pavé 2015). The gestation lasts six months; so on average the age of first reproduction could be around four-five years (Crockett & Eisenberg 1987, Treves 2001, Raguet-Schofield & Pavé 2015) or earlier (Glander 1980). To be conservative the age of first reproduction for females was set at five years in the baseline model and four years was set in the sensitivity analysis.

It was estimated that it would take longer for males to mate and reproduce since they must be able to secure a troop of females first. There is a lot of discussion in the literature regarding this parameter (Pope 1990). From observations in *A. caraya *(Agostini et al. 2012) and *Alouatta arctoidea* (Crockett & Eisenberg 1987, Rylands & Mittermeier 2013) the average age for first reproduction of males was set at six years and alternative value were tested in the sensitivity analysis (5 years) (Glander 1980, Moreland et al. 2001).

*Maximum age* - Vortex assumes that animals can reproduce (at the normal rate) throughout their adult life. Longevity was set as the maximum age of reproduction. A maximum of 25 years has been reported (Robinson & Redford 1986), but in general it is probably lower, around 16 years (Glander 1980, Pope 1990).

*Female breeding success* - According to Strier et al. (2001), the average inter-birth interval was 21.2 months. Therefore, it was considered that adult females produce one infant every two years. Different studies report different annual birth rates; the highest is recorded by Miranda (2004) with 0.72 infants per adult female per year. For this reason the percentage of female breeding was set on 50%, but can be as low as 28% and as high as 72%, which gives a SD of 22% (Table I).

*Maximum number of offspring per year* - *Mortality rates -*According to Strier et al. (2001), 74% of brown howler monkeys in the study survived their first year of life. Other studies seem to confirm this data (e.g., Pavé et al. 2012) and a mortality rate of 25% was set for both males and females from age zero-one. From the ages one-three the mortality rate tends to decrease (Raguet-Schofield & Pavé 2015). So, assumptions were made and set at 15% (age 1-2) and 10% (age 2-3). Between ages three-four they become sub-adults and mortality rates tend to drop (Agostini et al. 2012, Raguet-Schofield & Pavé 2015). However, males aged four years old tend to disperse more than females (Oklander et al. 2010) which can increase their chance of mortality (Otis et al. 1981). As both males and female sub-adults get older, mortality rates decrease (Raguet-Schofield & Pavé 2015). Mortality rates of adult howler monkeys are very low (Miranda 2004). However, it was estimated that for both males and females the mortality rate would increase after the age of 10.

For females after 10 years of age was estimated that mortality rates will increase by 5% and for males by 20%, each year. This is because after 10 years of age, the likelihood of the male having been expelled from his group increases (Pope 1990) and, if so, his mortality rate increases significantly.

For each mortality rate, alternative values based on SD were set to simulate the possible impact caused by the EV (Table I). Thus, more vulnerable ages, like the earlier and advanced stages of life, had higher values of SD estimated when compared with less vulnerable ages.

*Population description* - *Number of populations* - For the purpose of the baseline model one population was considered, but in reality, is composed of several smaller fragmented populations with unknown connectivity among them (see *Metapopulation simulation*).

*Initial population size (n)* - According to a best guess the population of Misiones could be around 200 individuals (Holzmann et al. 2010).

*Carrying capacity (K)* - Because we did not find a quantitative estimation of K specific for *Alouatta* sp., in this study we define K as a little more than double the initial population (n = 420). This is an assumption, similar to that made by Mandujano and Escobedo-Morales (2008). No EV was added to the K, as variations in population size are accounted for by EV in reproduction and survival.

*Number of catastrophes* - Catastrophes are singular environmental events that are outside of the bounds of normal EV affecting reproduction and/or survival. Natural catastrophes can be tornadoes, floods, droughts, disease or similar events. These events are modelled in Vortex by assigning an annual probability of occurrence and a pair of severity factors describing their impact on survival (across all age-sex classes) and the proportion of females successfully breeding in a given year. These factors range from 0 (maximum or absolute effect) to 1 (no effect) and are imposed during the single year of the catastrophe, after which time the demographic rates can rebound to their baseline values. In this sense, a simulation was conducted using the Vortex model alone, to compare the outcome of baseline model in scenarios with and without YF outbreaks. For this specific analysis, YF was set as a potential catastrophe (Crockett 1998) and an inter-epidemic interval of 15 years on average for Argentina (Di Bitetti et al. 1994, Camara et al. 2011) was converted into a 6% probability of occurrence of a YF outbreak each year. However, for the metamodel simulation, no catastrophes were set in Vortex and different probabilities of YF occurrence were defined by the sensitivity analysis using Outbreak (see*Contact with and transmission of virus through an external environmental source).*

*Harvest* - No harvest was included in the baseline model.

*Supplementation - *No supplementation of individuals from other unrelated populations, wild or captive, was incorporated into the baseline model.

A sensitivity analysis specific for the severity and frequency of YF outbreaks was performed in Vortex. Sensitivity to severity of the outbreak was modelled and mortality due to YF ranged from 90-20%. A frequency of 10-1% probability of an outbreak occurring each year was tested (maintaining the severity used in the base line model, killing between 50-80% of the population during an epidemic).

**Fig. 1A:**
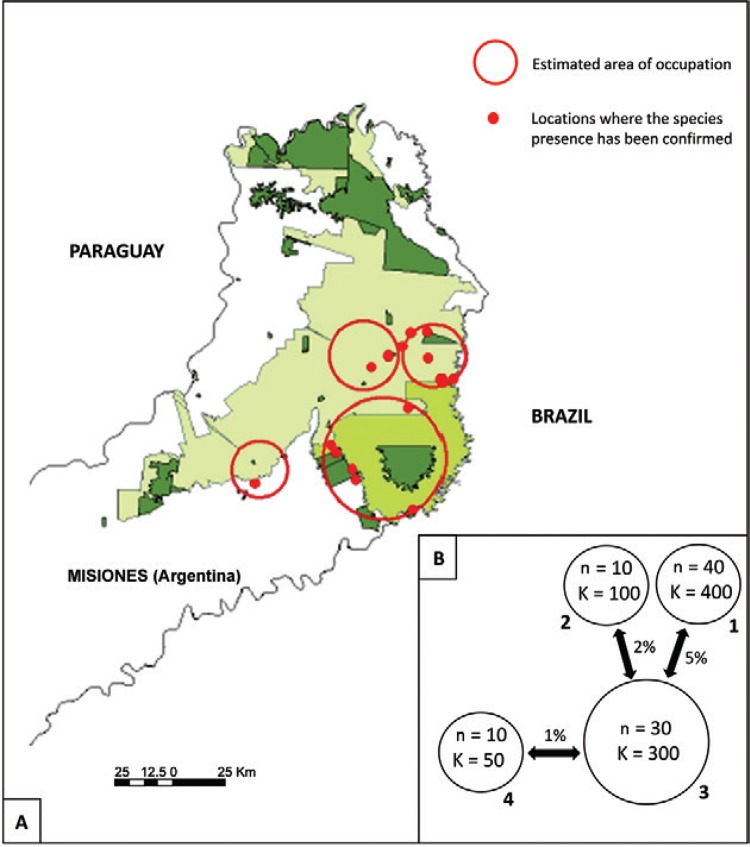
distribution of remnant populations of brown howlers in Misiones, Argentina, estimated by the workshop participants. Red circles represent the potential subpopulations (numbered from 1-4) currently present in Misiones. Red points represent locations where the species presence has been confirmed (at least before the yellow fever outbreaks); B: distribution, size (n), potential carrying capacity (K) and connectivity of brown howler monkeys estimated in Misiones by the workshop participants.

*Metapopulation simulation* - Due to forest fragmentation the remnant brown howler monkey population is becoming increasingly structured into subpopulations and so, might not be impacted by YF outbreaks in the same way. Thus, we tried to simulate how the population of brown howler monkey might potentially be distributed in Misiones (Fig. 1A), as well as what population sizes would be (n) and could potentially be (K) (Fig. 1B). There are no specific references to support these population numbers and were based on the field experience of researchers (Agostini et al. 2010, 2012, Holzmann et al. 2010). But, we believe, this simulation would be useful to illustrate the possible mechanisms related to metapopulation dynamics, not evaluated in the baseline model. Dispersal between populations was estimated and modelled. We estimated that males dispersed more than females (Oklander et al. 2010) and dispersal probably occurs between four-six years of age. Dispersal rates varied between fragments based on the perceived degree of connectivity and no mortality was considered. Of the dispersing individuals, assumptions were made based on Oklander et al. (2010), in which 70% were males and 30% females. Dispersal rates between populations are represented in Fig. 1B. For more details about methods for metapopulation input parameters in Vortex see Lacy et al. (2013) and Lacy and Pollak (2013).

*Input parameters for stochastic disease epidemiology models* -*Outbreak* simulates SEIR-style disease dynamics, which means that this model calls for an encounter rate between individuals (as the mechanism for direct transmission). To make the model able to support the transmission of a vector-borne disease, we translated some basic components (like exposed and infectious) into different ways of viewing the system: two concepts of the incubation period for the exposed state and two concepts of the Infectious period for the infectious state are described. First, as a simplified case, we considered only the (incubation and infectivity) period for the vertebrate host. In the second system, we selected the time needed to complete the full virus cycle, i.e., the incubation period in *Alouatta* individuals added to the extrinsic incubation period in the mosquito. For more details about the conceptual basis for adapting directly transmitted disease to mosquito-borne disease systems see Adams and Kapan (2009) and Manore et al. (2014).

*Pre-susceptible state* - We assume that all newly-born individuals become susceptible to YF immediately after birth, except in cases when a mother has recovered from infection with the pathogen. In that case, newborn individuals acquire temporary immunity from infection with YF (CDC 2010, Traiber et al. 2011). This temporary immunity lasts between one-180 days (lactation period) for each newborn animal, with a uniform probability of reverting to the susceptible state during this period of time (Johansson et al. 2010, Moreno et al. 2011, Traiber et al. 2011).

*Susceptible state* - *Encounter rate* - Considering that YFV is a vector-transmitted pathogen with multiple potential hosts and vectors, the traditional concept of contact rates among diseased host individuals does not fit in our case. One infected NHP can infect even hundreds mosquitoes and the infection can quickly spread within a population, showing a multiplicatory effect (Consoli & Lourenço-de-Oliveira 1994,Vasconcelos 2010). Therefore, it is impossible not to consider the encounter rate as a function of a combination of factors: (i) mosquitoes dispersal ability, (ii) host and vector densities, (iii) the number of blood-feeding events where howler monkeys are used as food source and (iv) the number of blood-feeding events where other potential mammalian are used as food source.

These parameters are highly uncertain in the scientific literature. Therefore, in order to identify the importance of this variable in the modelling process, we set minimum and maximum values of encounter rates as 1:1 and 1:20, i.e., one viraemic mosquito can transmit the disease from a minimum of one to a maximum of 20 individuals. Then, we performed a sensitivity analysis using this range of values.

*Transmission rate* - We considered this variable to be a function of the natural infection rate of YFV in mosquitoes. We set a value of 0.2 for transmission rate given contact; in other words, a 20% chance that a viraemic mosquito will successfully transmit YFV when biting a naïve howler monkey. For the sensitivity analysis we chose alternative values of 0.5 and 0.8.

*Contact with and transmission of virus through an external environmental source *- To estimate this parameter value, we noted that the YF shows a cycle with a minimum of approximately 14 years (5,110 days) between outbreaks as reviewed by Camara et al. (2011) for the South Region of Brazil. This may be considered a true minimum value, since in Argentina the last YFV transmission recorded was in 1967 (Goenaga et al. 2012). In this analysis, the specific inter-epidemic interval for Argentina was assumed, for the baseline model, in the order of 30 years (10,950 days) (Di Bitetti et al. 1994). For the sensitivity analysis, shorter and extremely longer intervals were tested, 14 and 300 years, respectively. Therefore, the probability of acquiring the pathogen from an external environmental source is simply the reciprocal of the inter-epidemic interval (e.g., 1/inter-epidemic interval in days).

*Incubation period* - To estimate this parameter we considered two alternative ways. First, we considered only the incubation period for primates of the genus *Alouatta*. According to this criterion, the latent period would vary between three-six days (Anderson & Downs 1955, Johansson et al. 2010). As an alternative for the sensitivity analysis, we included the extrinsic incubation period in the mosquito (Mondet 1997, Johansson et al. 2010). This led to a latent period of 15-20 days. The sensitivity analysis, then, included these two values as alternative inputs.

*Infectious state* - *Infectious period* - We evaluated two alternative ways to estimate the infectious period parameter. First, we considered the period of viraemia in *Alouatta* individuals using data available from the literature, i.e., three-six days (Anderson & Downs 1955, Johansson et al. 2010). Second, we selected as the infectious period the time it takes for an infected mosquito to transmit the disease (Forattini 2002). Assuming that an infected mosquito can transmit the disease during its entire life span, the researchers therefore estimated the maximum duration of the infectivity period as 30-60 days (Mondet 1997). These two alternatives were used as inputs for the sensitivity analysis.

*Disease outcome* - No infectious individual host remains in the infectious state indefinitely; all animals either clear the infection after 10-20 days of time or die (Anderson & Downs 1955, Johansson et al. 2010). Individuals that survive the infection develop permanent immunity to future infective events (Poland et al. 1981, Schlesinger et al. 1986). Therefore, the probability of surviving individuals returning to the susceptible state would be zero. We simulated three different mortality scenarios as part of the epidemiological sensitivity analysis: a mild event characterised by 20% mortality of infected animals, a medium-level event characterised by 50% of mortality and a severe event with 80% of mortality.

*Recovered state* - *Permanent resistance* - As indicated by studies of Poland et al. (1981), an animal that recovers from an infection with the YFV is permanently immune from further infection, the proportion of recovered individuals that acquire permanent immunity in our models was set to 1.0.

Table II presents a summary of the input parameters used for the Outbreak model.

**TABLE II:** Input parameters used in the Outbreak models of yellow fever (YF) epidemiology in brown howler monkeys of Argentina

Parameter	Baseline value	Alternate values
Pre-susceptible		
Newborns with permanent immunity	0.0	-
Duration of maternally-derived immunity (days)	180	-
Susceptible		
Encounters per day	10	1, 20
Transmission rate given encounter	0.2	0.5, 0.8
Encounter rate with outside source^*a*^	9.1 x 10^-5^	2 x 10^-4^, 9.1 x 10^-6^
Transmission rate given external encounter	0.2	0.5, 0.8
Exposed		
Incubation period (days)	3-6	15-20
Infectious		
Infectious period (days)	3-6	30-60
Probability of recovering to susceptible state	0	-
Probability of recovering to resistant state	0.5	0.2, 0.8
Probability of dying from the infection	0.5	0.2, 0.8
Recovered/resistant		
Proportion acquiring permanent immunity	1	-

*a*: between the howler monkey population and an outside source of YF virus. See accompanying the main text for detailed explanation of input parameter definitions.

To perform sensitivity analyses, 15 model scenarios were constructed, each with a specific parameter value changed from its baseline model value according to the information presented in Table II. In each of these scenarios, only one parameter value was changed, with all other input parameters set at their baseline values.

*Disease dynamic simulation* - To better illustrate the single iterations in the YF disease dynamics, we made a simulation using Outbreak alone. For this, we set a starting population of 30 brown howler monkeys, aged one year and older. Furthermore, for the purpose of this modelling exercise, we assumed that this population occupies a habitat area that has a K of 50 individuals through time. Each scenario featured 500 iterations and was run for 100 years.

## RESULTS

*Baseline model* - Vortex model featuring no YF outbreaks showed a positive stochastic population growth rate (0.026) and the population could potentially increase almost 3% a year. The stochastic growth rate with YF as a catastrophe was negative (-0.051); this means that according to the model using Vortex alone, the population of Misiones has a 20% of chance of survival in 100 years due to recurrent YF outbreaks.

The baseline Vortex howler monkey demographic model, linked to the baseline Outbreak model of YF epidemiology, showed an annual stochastic population growth rate of -0.045 and the risk of population extinction by the end of the simulation was even higher (98%).

**Fig. 2A:**
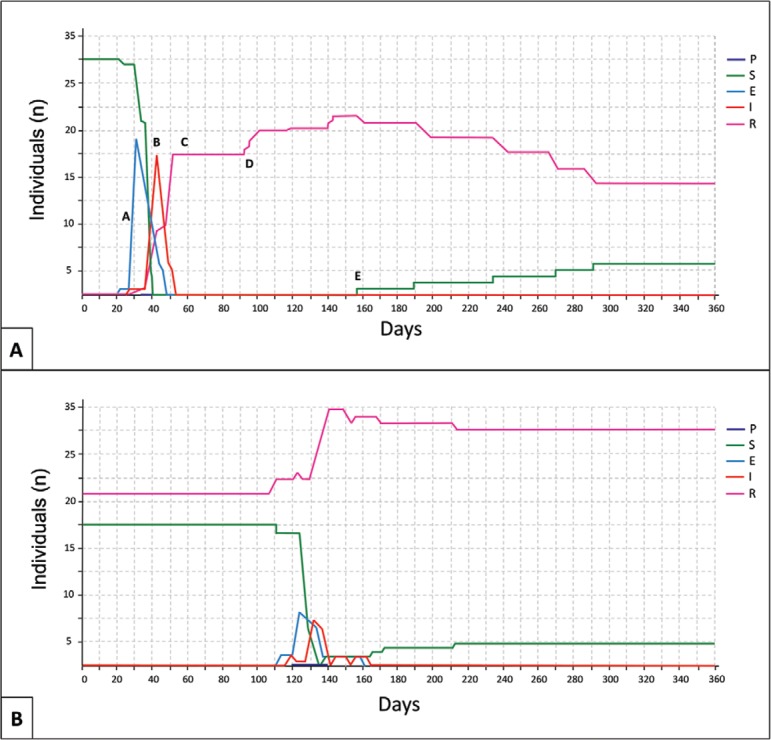
trajectory of disease dynamics in year 1 of a single iteration of the baseline brown howler monkey simulation model using Outbreak alone. Specific points in the trajectory are designated by letters A-E and are described in more detail in the main text; B: trajectory of disease dynamics in year 50 of a single iteration of a brown howler monkey simulation model using Outbreak alone. Note the relatively high proportion of resistant individuals at the start of the outbreak on day 115, leading to a yellow fever outbreak of lower overall intensity. E: exposed; I: infectious; P: pre-susceptible; R: recovered/resistant; S: susceptible.

We can better understand the dynamics of disease by looking in detail at the Outbreak model output for single iterations. For example, in the first outbreak episode of a given model iteration (Fig. 2A), a rapid increase in exposure to the pathogen (point A on the graph) is quickly followed by a similar increase in the proportion of infected individuals in the population (point B). Because we specified approximately 50% disease-based mortality in our baseline model, exposure and infection are quickly followed by a reduction in overall population abundance by approximately 50% with all of those surviving individuals being resistant to further infection (point C). Newborn individuals with maternally-derived immunity to the disease begin appearing about day 92 (point D) and gradually lose that immunity and begin transitioning to the susceptible state beginning on day 155 (point E). As time progresses in the simulation and the number of surviving and resistant individuals begins to increase, the intensity of the outbreak is reduced (Fig. 2B).

**Fig. 3:**
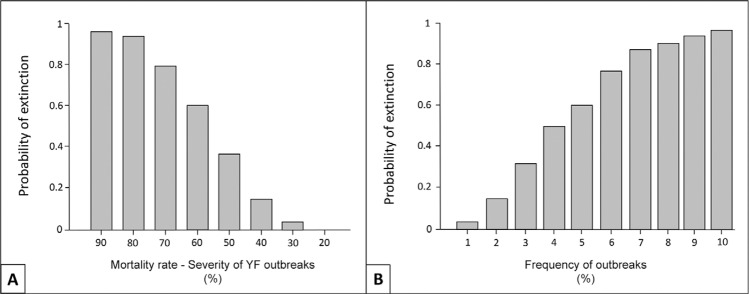
probability of extinction according to the severity of yellow fever (YF) outbreaks (A) and according to the frequency of outbreaks (B).

*Sensitivity analysis* - Both severity and frequency of YF have an impact on the rate of extinction of brown howler monkeys. The change in survival rates after each outbreak had more impact than frequency of outbreaks. Therefore, the impact of an YF outbreak in a population is devastating, as the mortality rate increases (Fig. 3).

**Fig. 4:**
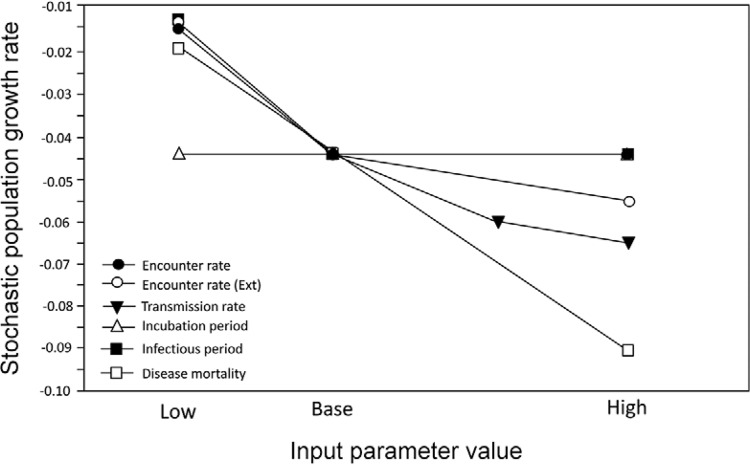
epidemiological sensitivity analysis of a simulated population of brown howler monkeys subject to yellow fever (YF) outbreaks. Those curves with the steepest slope indicate the model parameters with the greatest overall sensitivity. Ext: between the howler monkey population and an outside source of YF virus.

However, because of the relatively severe 50% mortality impact of any outbreak as defined in our baseline model, even a low outbreak frequency leads to a reduced growth rate and increased extinction risk. In a similar fashion, increasing the transmission rate (Table III) following contact has a major impact on the simulated population, leading to a significant reduction in population growth rate (Fig. 4).

**TABLE III:** Input parameter values and model results for epidemiological sensitivity analysis using the linked Vortex - Outbreak metamodel discussed in the main text

Scenario	r_s_	P(E)	T(E)	N_100_
Baseline	-0.045	0.988	46.5	0.06
Fixed encounter: 1 individual/day	-0.014	0.486	75.4	8.81
Fixed encounter: 20 individuals/day	-0.045	0.992	47.3	0.05
Encounter rate with external source: 9.1 x 10^-6^	-0.015	0.542	72.0	7.70
Encounter rate with external source: 2.0 x 10^-4^	-0.058	0.998	37.2	0.02
Transmission rate given encounter: 0.5	-0.060	1.000	35.9	-
Transmission rate given encounter: 0.8	-0.066	1.000	32.9	-
Incubation period: 15-20 days	-0.046	0.992	46.9	0.05
Infectious period: 30-60 days	-0.047	0.996	46.5	0.07
Disease mortality: 0.2	-0.021	0.630	73.9	4.85
Disease mortality: 0.8	-0.092	1.000	21.4	-

N_100_: mean population size that would survive over a 100 year period; P(E): probability that the population will go extinct in a 100 year period; r_s_: mean rate of stochastic population growth or decline; T(E): mean time to population extinction in years.

In the baseline model, YF outbreaks occurred approximately 10-14 times in the 100-year simulation. When encounter rate is reduced, both within the population of howler monkeys and between howler monkey populations and an outside source of YFV, the number of outbreaks drops to just one or two events during the 100 years. With a constant encounter rate but a higher transmission rate, the frequency of outbreaks and the probability that the population will go extinct, increases significantly (Fig. 4, Table III).

**Fig. 5:**
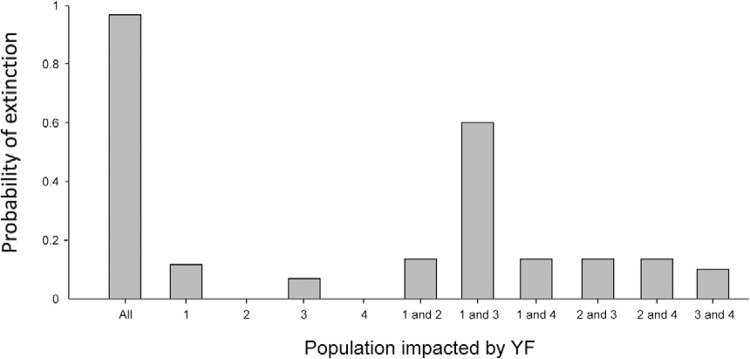
probability of extinction when yellow fever (YF) outbreaks hit all or only some populations.

*Metapopulation simulation* - According to the model, YF does not impact all fragments equally (Fig. 5). When all populations are impacted by the same YF outbreak, the probability of extinction is 98%. However, if the outbreak only hits one or two of the populations, then the probability of extinction decreases. Probability of extinction was highest when the YF outbreak impacts the largest populations (1 and 3) simultaneously.

## DISCUSSION

There is a significant amount of measurement uncertainty associated with many of the input parameters used in our Vortex and Outbreak models of YF disease epidemiology. Nevertheless, an analysis of the sensitivity of our models to this measurement uncertainty can be a valuable aid in identifying priorities for detailed research and/or management projects targeting specific elements of disease epidemiology and/or ecology.

The baseline model in Outbreak was shown to be highly useful to estimate and visualise the different stages of transition in a howler population impacted by an YF outbreak.

In the metapopulation simulation analysis, we illustrated that YF does not impact all fragments equally and the probability of extinction was reduced compared with the concentrated population analysed in the baseline model. These findings corroborate those of Crockett (1998) who cites that fragmentation of forests may actually reduce YF transmission and management programs could reduce probability of extinction of howler groups through reintroductions or translocations in most impacted forest fragments.

The sensitivity analysis showed that the frequency and impact of outbreaks is strongly affected by the encounter rate (Fig. 4, Table III), both within the population of howlers and between the howler populations and an outside source of YFV.

The sensitivity analysis also demonstrates that adult mortality has one of the highest impacts on the population. Current long term studies (Strier et al. 2001, Miranda 2004) show that adult mortality is extremely low in healthy howler monkey populations. Therefore, the impact of an YF outbreak on a population is devastating, as the mortality rate highly increases (Fig. 3). According to Crockett (1998), the higher reproductive potential of *Alouatta* than expected for its body size would be characteristics of *Alouatta*'s opportunistic habits or adaptations to its high susceptibility to YF.

However, even the simulation models featuring low lethality rates tended to result in local population extinction. In this case, periodic epidemics might cause the population to decline to levels that lead to inbreeding depression, such that the elevated probability of extinction under these low lethality scenarios might not have occurred if inbreeding depression was not in the model.

There could be a complex interplay between inbreeding and disease (Spielman et al. 2004) and more studies have to be conducted to determine the precise relationship between inbreeding depression and YF (Pope 1996, Crockett 1998).

In contrast to these highly sensitive parameters that strongly affect transmission dynamics and disease-based mortality, other parameters do not seem to influence long-term disease dynamics or population demographic stability. Specifically, changing the baseline values for the incubation (latent) period of the pathogen and the infectious period lead to only very small impacts on population outcome. So these should not be a priority for research.

Predicting patterns of mosquito spread is complicated by complexity of the systems, lack of appropriately granular data and computational expense of realistic models (Manore et al. 2014). In this sense, modelling approaches using indirect indicators to access mosquito dynamics has gained visibility to understand complex mosquito-borne diseases problems (Adams & Kapan 2009, Perkins et al. 2013, Stoddard et al. 2013, Manore et al. 2014).

Despite the Outbreak software had not be directly structured to deal with vector borne disease models, the adaptations made for these two components showed to have few impacts in the whole model performance. Adams and Kapan (2009) were able to show that host behaviour and movement through the mosquito environment can be important and perhaps more crucial to understanding risk and informing mitigation efforts in vector-borne diseases.

The analyses presented here represent one of the first detailed applications of a metamodel linking the well-known population viability analysis software package Vortex to a sophisticated model of infectious disease epidemiology, Outbreak.

The approach showed in this study can be also used as a tool for disease control, being useful to make projections about impacts on the species conservation with direct repercussions on human health. Models like the one depicted here can constitute important tools to guide decision-makers in YF outbreak emergency situations.
